# The metabolomic profile of gamma-irradiated human hepatoma and muscle cells reveals metabolic changes consistent with the Warburg effect

**DOI:** 10.7717/peerj.1624

**Published:** 2016-01-26

**Authors:** Min Wang, Adrian Keogh, Susan Treves, Jeffrey R. Idle, Diren Beyoğlu

**Affiliations:** 1 Institute of Integrated TCM and West Medicine, Medical College, Lanzhou University, Lanzhou City, Gansu Province, P.R. China; 2 Hepatology Research Group, Department of Clinical Research, University of Bern, Bern, Switzerland; 3 Visceral and Transplantation Surgery, Department of Clinical Research, University of Bern, Bern, Switzerland; 4 Departments of Anesthesia and Biomedicine, University Hospital Basel, Basel, Switzerland

**Keywords:** Gamma-irradiation, HepG2 cells, HMCL-7304 myotubes, Metabolomics, GCMS, Warburg effect

## Abstract

The two human cell lines HepG2 from hepatoma and HMCL-7304 from striated muscle were γ-irradiated with doses between 0 and 4 Gy. Abundant γH2AX foci were observed at 4 Gy after 4 h of culture post-irradiation. Sham-irradiated cells showed no γH2AX foci and therefore no signs of radiation-induced double-strand DNA breaks. Flow cytometry indicated that 41.5% of HepG2 cells were in G2/M and this rose statistically significantly with increasing radiation dose reaching a plateau at ∼47%. Cell lysates from both cell lines were subjected to metabolomic analysis using Gas Chromatography-Mass Spectrometry (GCMS). A total of 46 metabolites could be identified by GCMS in HepG2 cell lysates and 29 in HMCL-7304 lysates, most of which occurred in HepG2 cells. Principal Components Analysis (PCA) showed a clear separation of sham, 1, 2 and 4 Gy doses. Orthogonal Projection to Latent Structures-Discriminant Analysis (OPLS-DA) revealed elevations in intracellular lactate, alanine, glucose, glucose 6-phosphate, fructose and 5-oxoproline, which were found by univariate statistics to be highly statistically significantly elevated at both 2 and 4 Gy compared with sham irradiated cells. These findings suggested upregulation of cytosolic aerobic glycolysis (the Warburg effect), with potential shunting of glucose through aldose reductase in the polyol pathway, and consumption of reduced Glutathione (GSH) due to γ-irradiation. In HMCL-7304 myotubes, a putative Warburg effect was also observed only at 2 Gy, albeit a lesser magnitude than in HepG2 cells. It is anticipated that these novel metabolic perturbations following γ-irradiation of cultured cells will lead to a fuller understanding of the mechanisms of tissue damage following ionizing radiation exposure.

## Introduction

There is an ever increasing threat from the terrorist use of nuclear isotopes. To date, the only intentional exposure of humans to ionizing radiation from a nuclear device occurred during the first use of the atomic bomb at Hiroshima and Nagasaki on 6 and 9 August 1945, respectively. Accidental exposures have also occurred. Ten persons were subjected to bursts of ionizing radiation at the Los Alamos Scientific Laboratory in accidents that occurred on 21 August 1945 and 21 May 1946 (Hempelmann, Lisco & Hoffman, 1952). The estimated median combined X-ray and γ-ray dose to these subjects was 2.7 Gy (range 0.27 to 18 Gy). The two highest exposed persons died while the others made a full recovery (Hempelmann, Lisco & Hoffman, 1952). These unfortunate accidents provided the first opportunity to examine both blood and urine for the footprints of high-dose radiation exposure. The blood chemistry provided no results that were either specific or of significance. In contrast, the subjects who had received the highest doses of radiation exhibited urinary concentrations of amino acids in the range 1 to 2 mg/ml, most notable of which was the excretion of taurine (Hempelmann, Lisco & Hoffman, 1952). These human findings were soon replicated rats receiving 8 to 25 Gy X-irradiation (Kay & Entenman, 1954) and confirmed by others in the rat (Aebi et al., 1955) and in four human subjects accidentally exposed to γ-irradiation and neutrons at the Argonne National Laboratory (Katz & Hasterlik, 1955).

A rat model of acute radiation sickness was studied in the former USSR. Administration of 2 or 4 μCi ^90^Sr to rats caused a marked increase in the urinary excretion of thymidine (Uspenskaia & Rabinkova, 1965), with 8-fold increases reported in another study (Zharkov, Fedorova & Mikhailova, 1965). Enhanced urinary excretion of the related nucleoside pseudouridine was observed in cancer patients receiving X-ray therapy and in rats receiving whole-body X-irradiation. The authors believed that the spleen contributed largely to these findings (Portmann et al., 2013). The early literature, therefore, described the elevated urinary excretion of both amino acids and nucleosides in humans and rats exposed to large doses of ionizing radiation.

This remained the case until the problem was readdressed using high-throughput metabolomic technologies in the recent past. The pattern described four to five decades earlier has essentially remained the same, but with more related metabolites added. Interestingly, both taurine and thymidine have persisted as in vivo urinary biomarkers of ionizing radiation exposure in the mouse, rat and rhesus monkey (Johnson et al., 2011; Johnson et al., 2012; Tyburski et al., 2008, Tyburski et al., 2009).

There has been a recent concerted effort to discover biomarkers of ionizing radiation exposure that could lend themselves to high-throughput minimally-invasive population screening in the event of intentional or accidental mass exposures to radiation. Metabolomics has been part of this effort (Johnson et al., 2011; Johnson et al., 2012; Laiakis, Hyduke & Fornace, 2012; Laiakis et al., 2014; Lanz et al., 2009; Manna et al., 2013; Tyburski et al., 2008, Tyburski et al., 2009). Despite the uncovering of radiation biomarkers, little, if anything, is known about their cellular origins. Irradiation of cells in culture could help fill this knowledge gap.

Irradiation of cultured cells has a long history, beginning with chick embryo fibroblasts given X-ray doses of 1 to 10 Gy in an attempt to improve the efficacy of X-ray therapy with radiosensitizers (Mitchell & Simon-Reuss, 1952). More recently, both human fibroblast and lymphoblast cell lines have been γ-irradiated to define biomarkers of ionizing radiation that might assist with human in vivo studies (Patterson et al., 2008). While clear metabolomic phenotypes were found for doses of between 0.5 and 8 Gy, the only identified metabolites were those that were depleted by cellular irradiation; for example, GSH and AMP. No positive biomarkers of ionizing irradiation were reported (Patterson et al., 2008). In an effort to redress this deficiency and to define biomarkers of exposure to ionizing radiation in human cells in culture, we have conducted a study of γ-irradiation of the human hepatocellular carcinoma cell line HepG2 and human skeletal muscle cell line HMCL-7304 using gas chromatography-mass spectrometry based metabolomics. Cell lines from liver and skeletal muscle were chosen because these tissues represent the major stores of taurine (Awapara, 1956), the historical biomarker for ionizing radiation (see above), and therefore perhaps are tissues that are sensitive to ionizing radiation.

## Materials and Methods

### Cell culture

HepG2 cells were supplied frozen by ATCC (LGC Standards GmbH, Wesel, Germany). Cells were cultured to 90% confluence in T75 culture flasks in DMEM containing 10% FBS and 100 U/ml penicillin/100 μg/ml streptomycin (Life Technologies, Carlsbad, CA, USA), as described (Portmann et al., 2013), then split 3:1 and subcultured in T75 flasks. HMCL-7304 human myotubes were derived from an immortalized myoblast cell line that had been established from the intercostal skeletal muscle of a female donor with no neuromuscular disorder (Rokach et al., 2013) and that had been maintained at the University of Basel. Cells were cultured in PromoCell skeletal muscle cell growth medium (Vitaris AG, Baar, Switzerland) in a low oxygen environment (5% O_2_ and 5% CO_2_), as described (Rokach et al., 2013). Differentiation to myotubes was induced by culture in PromoCell skeletal muscle differentiation medium (Vitaris) and, after 5 days, multinucleated myotubes were visible under low magnification and referred to as HMCL-7304 cells (Rokach et al., 2013).

### Gamma-irradiation of cells

Cell culture flasks containing 4 × 10^6^ cells were γ-irradiated in groups of six using a Gammacell 40 Exactor (Best Theratronics, Ottawa, Canada). The irradiator was fitted with two ^137^Cs sources (above and below) with an activity of 1800 Ci/67 TBq and delivering 1.0 Gy/min. Nominal radiation doses of 0, 1, 2 and 4 Gy were employed. Sham irradiation (0 Gy) was achieved by placing the culture flasks in the irradiator for 2 min without irradiation. Immediately after irradiation, cells were cultured for a further 4 h. One flask of HepG2 cells at each dose was used for FACS and fluorescence immunohistochemical (IHC) analyses and five flasks were used for metabolomic analysis. HMCL-7304 myotubes were subjected only to metabolomic analysis after irradiation.

### Determination of the cellular effects of γ-irradiation

It is necessary to obtain evidence that γ-irradiation produced characteristic changes to the cells at the doses employed. Therefore, HepG2 cell cultures that were submitted to metabolomic analysis were also analyzed for evidence of radiation damage by two methods. Firstly, flow cytometry was performed for the purposes both of cell counting and for evaluating the cell cycle stage of all cell cultures using a FACS LSR II flow cytometer (BD Biosciences, Allschwil, Switzerland). For cell cycle staging, propidium iodide staining was used after RNase I treatment of combined adherent and floating cells (Pang et al., 2013; Shabalina et al., 1999). Combined adherent and floating cells were counted by flow cytometry (Brunck et al., 2014) using BD Trucount absolute counting tubes (BD Biosciences). Secondly, cells were examined using IHC for γH2AX foci as evidence of DNA double strand breaks, as previously described (Zhao et al., 2013). Neither flow cytometry nor IHC was performed on HMCL-7304 myotubes.

### Gas chromatography-mass spectrometry based metabolomics

Adherent cells were removed by gentle scraping, washed twice with ice-cold PBS, spun, resuspended in ice-cold distilled water and briefly sonicated to liberate water-soluble metabolites as described (Patterson et al., 2008). Briefly, aqueous supernatants were first blown to dryness at 40 °C under a gentle stream of N_2_. Dry residues were derivatized first with methoxyamine hydrochloride and then BSTFA containing 1% TMCS (N,O-bis[trimethylsilyl]trifluoroacetamide/trimethylchlorosilane) and samples analyzed by Gas Chromatography-Mass Spectrometry (GCMS) as described (Fahrner et al., 2012; Semmo et al., 2015), with data collected at retention times 13 to 63 min. QC samples, prepared from aliquots of each cell lysate, were included and analyzed as the first five samples injected and then interspersed between every fifth analytical sample.

Two independent criteria were used for the identification of HepG2 and HMCL-7304 cellular metabolites. Firstly, the mass spectra of each peak in the chromatograms were compared with a spectral library that contained 276,248 electron-impact mass spectra from 242,466 compounds (NIST 14 Mass Spectral Library, MS Wil GmbH, Wil, Switzerland). Only spectral matches of >90% were accepted for provisional identification. Secondly, mass spectra and retention times of each peak were compared with those from an in-house library of >100 authentic compounds that are found commonly in human biofluids and cell lysates. Spectral comparisons and retention times matches within ±0.05 min were accepted for peak annotation. Finally, peaks that were present in solvent and water blanks, such as glycerol monopaltimate and monostearate, which appeared to derive from laboratory plasticware, were neither quantitated nor included in the metabolomic analysis. Leaching of lipids molecules from laboratory plasticware has been described (McDonald et al., 2008).

Relative concentrations of each identified peak were derived, first using AutoQuant in the onboard Agilent software ChemStation, and then with QuantBrowser GCMS software (Leoson BV, Middelburg, The Netherlands) using the Peak Area Ratio (PAR) to the internal standard 4-chlorophenylacetic acid, present in each chromatogram as its trimethylsilyl ester. The resulting spreadsheet of PARs for each annotated metabolite was imported into SIMCA 13 (Umetrics, Malmö, Sweden) for multivariate data analysis using unsupervised Principal Components Analysis (PCA), supervised Projection to Latent Structures-Discriminant Analysis (PLS-DA) and Orthogonal PLS-DA (OPLS-DA), as described (Semmo et al., 2015).

## Results

**Figure 1:**
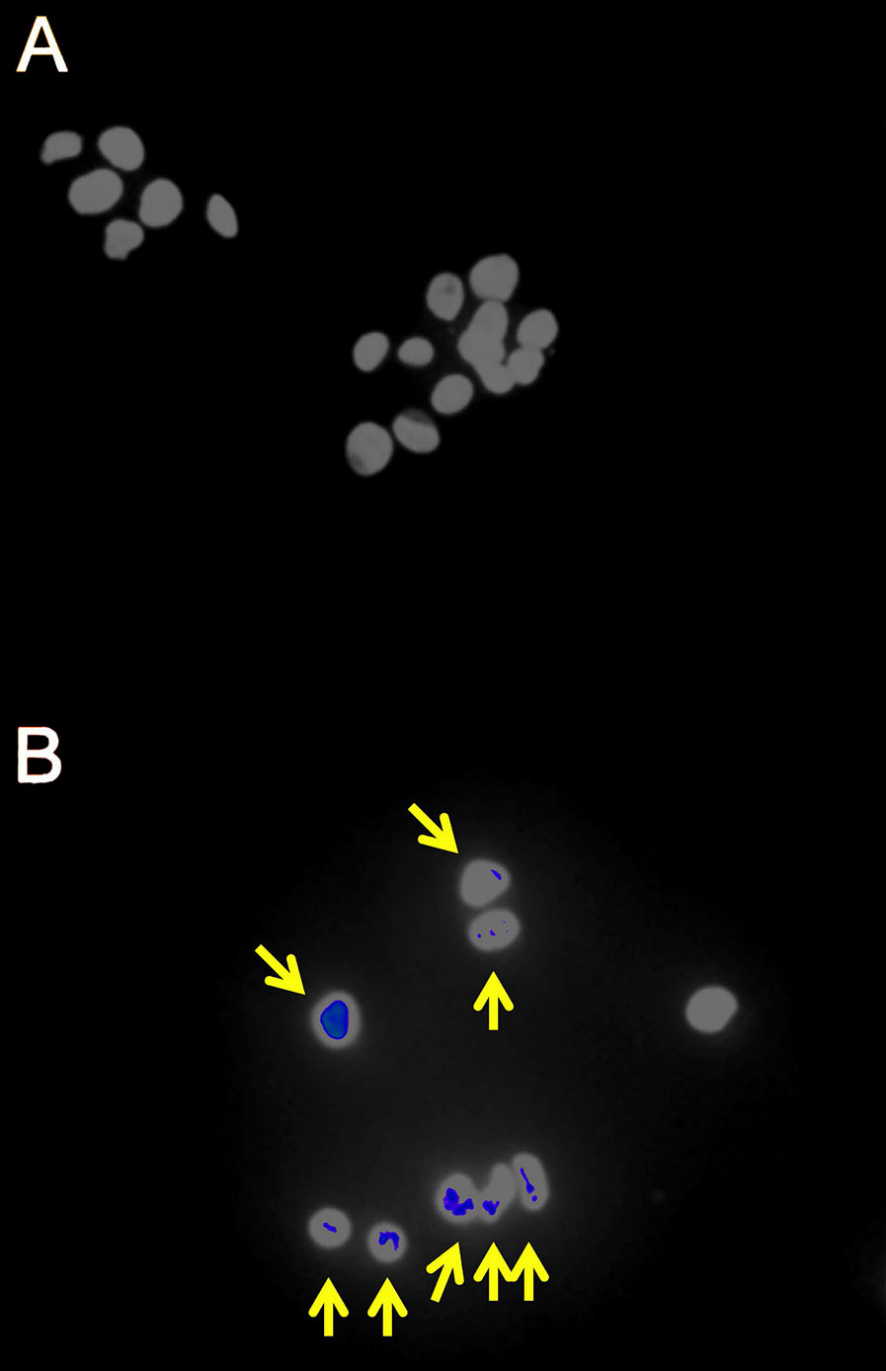
Fluorescence immunohistochemistry for γH2AX foci in γ-irradiated HepG2 cells. (A) Sham irradiated HepG2 cells showing no γH2AX foci after 4 h culture and therefore no signs of DNA damage. (B) HepG2 cells γ-irradiated with 4 Gy showing γH2AX foci (yellow arrows) after 4 h culture and therefore DNA double-strand breaks.

**Figure 2:**
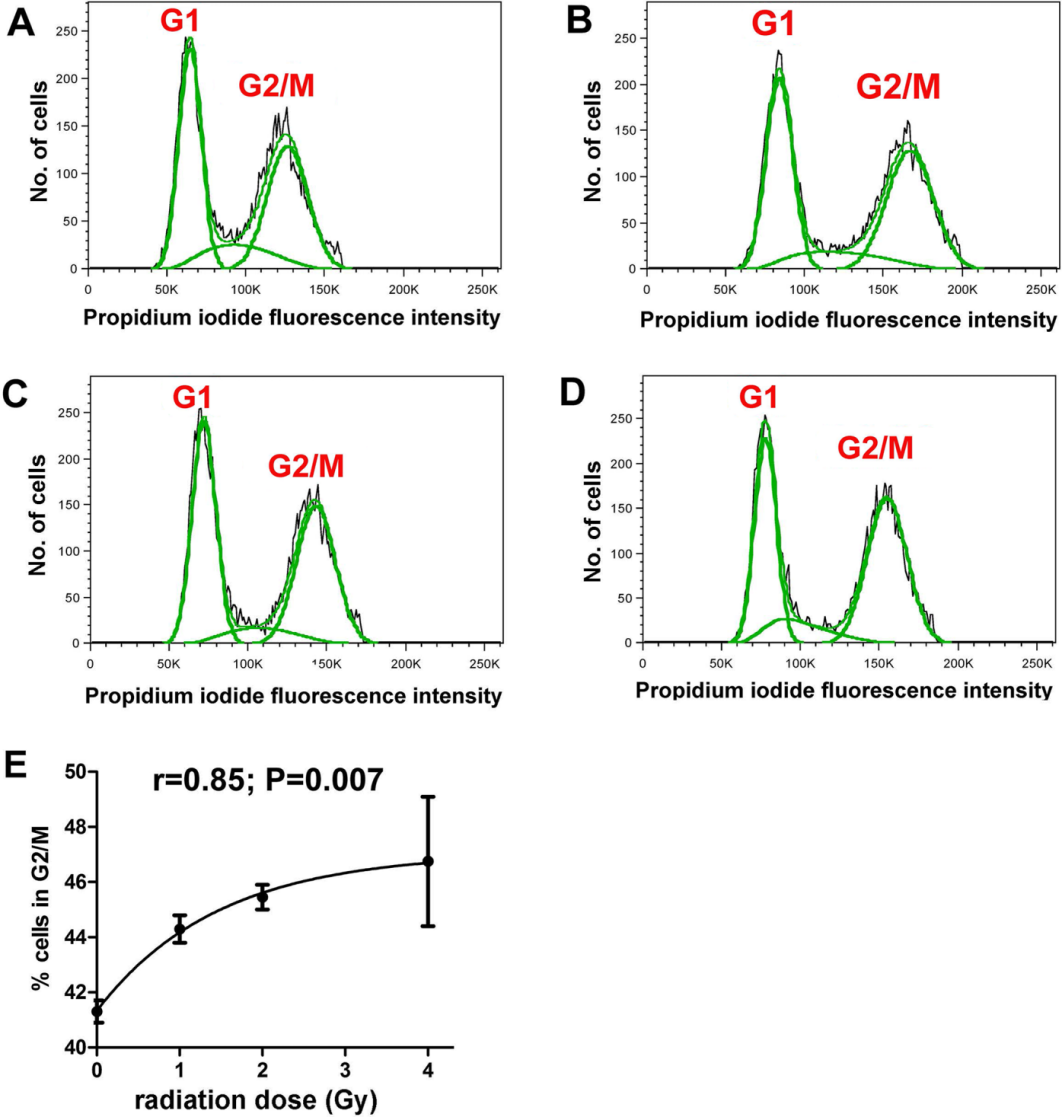
Cell cycle analysis of γ-irradiated HepG2 cells. Flow cytometry of HepG2 cells after different doses of γ-irradiation. (A) Sham irradiated. (B) 1 Gy irradiation. (C) 2 Gy irradiated. (D) 4 Gy irradiated. (E) Relationship between radiation dose (Gy) and proportion of cells in G2/M. There occurs a slight and saturable shift from G1 to G2/M with increasing dose of γ-irradiation.

It was first demonstrated that the radiation doses employed produced measurable cellular changes in HepG2 cells. Fluorescence IHC showed that γ-irradiated cells, but not sham irradiated cells, had multiple γH2AX foci indicative of DNA double-strand breaks (Fig. 1). In addition, flow cytometry analysis of sham and γ-irradiated HepG2 cells showed roughly equal populations of cells in G1 and G2/M stages of the cell cycle (Figs. 2A–2D). Although the changes were relatively small, the cells shifted significantly (r = 0.85; P = 0.007) from G1 to G2/M in relation to radiation dose, plateauing at ∼47% in G2/M (Fig. 2E).

**Figure 3:**
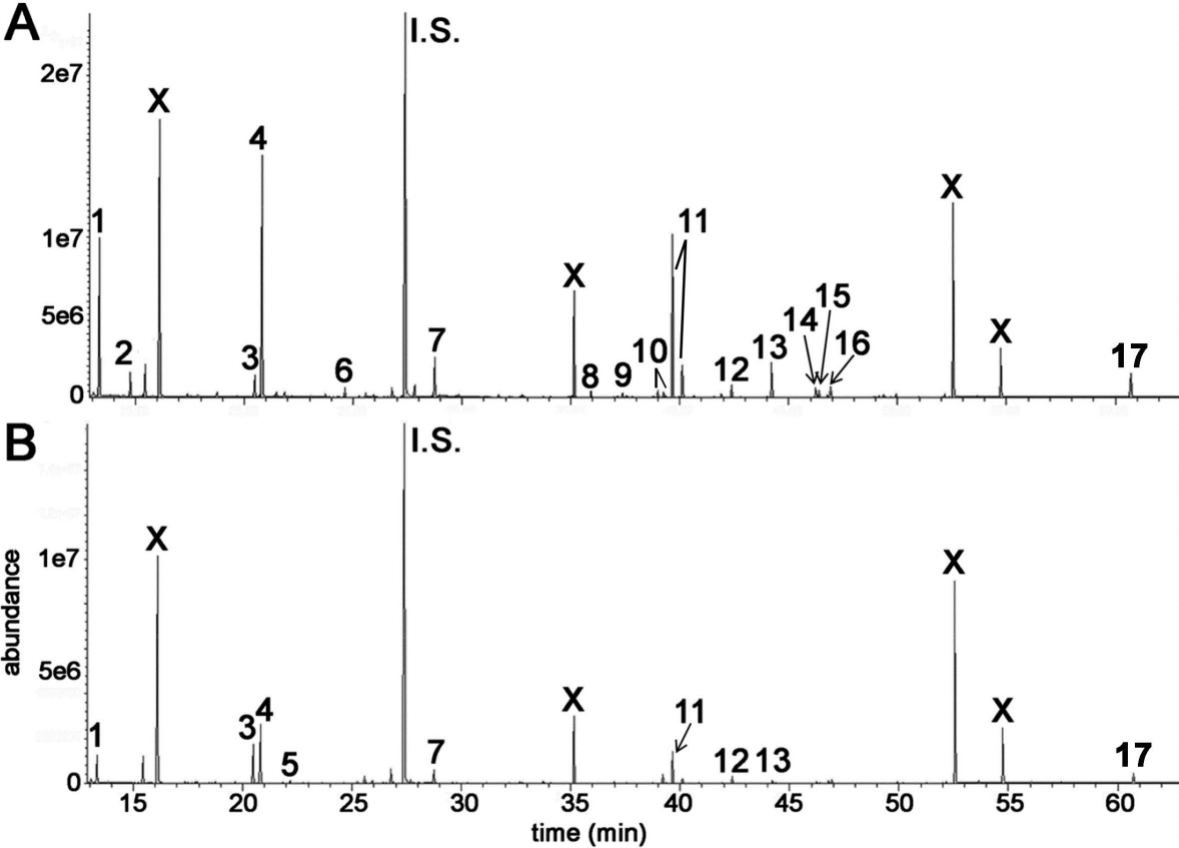
GCMS chromatograms for irradiated HepG2 hepatocellular carcinoma cells and HMCL-7304 myotubes. Cell lysates after γ-irradiation were derivatized with BSTFA/TMCS and MOX (see text). (A) HepG2 cell chromatogram. (B) HMCL-7304 myotube chromatogram. I.S. means internal standard (4-chlorophenylacetic acid). 1 = lactic acid; 2 = L-alanine; 3 = ethanolamine; 4 = phosphate; 5 = glyceric acid; 6 = L-threonine; 7 = L-5-oxoproline; 8 = glycerol 3-phosphate; 9 = citric acid; 10 = D-fructose; 11 = D-glucose; 12 = palmitic acid; 13 = *myo*-inositol; 14 = oleic acid; 15 = vaccenic acid; 16 = stearic acid; 17 = cholesterol; X = background contaminating peaks from reagents and laboratory plasticware.

GCMS was used to conduct metabolomic analyses on both HepG2 human hepatocellular carcinoma cells and HMCL-7304 human striated muscle myotubes. A total of 46 compounds were identified unequivocally in γ-irradiated HepG2 cell lysates, using both library spectra (>90% match) and a collection of more than 100 authentic standards. These HepG2 cell metabolites are listed in Table 1 and the most prominent 17 are shown in Fig. 3A. In the case of γ-irradiated HMCL-7304 myotubes, the resulting peaks (Fig. 3B) were smaller and, as Table 2 shows, it was only possible to identify 29 metabolites, all of which were also present in the HepG2 cell lysates.

**Table 1:** Metabolites detected by GCMS in lysates of HepG2 cells.

Metabolite	Retention time (min)	Derivatives formed	HMDB number
L-Lactic acid	13.34	2TMS	00190
L-Alanine	14.76	2TMS	00161
Glycine	15.33 21.85	2TMS 3TMS	00123
L-Valine	18.74	2TMS	00883
L-Leucine	20.68	2TMS	00687
Phosphate	20.82	3TMS	01429
L-Isoleucine	21.43	2TMS	00172
L-Proline	21.50	2TMS	00162
Succinic acid	21.99	2TMS	00254
Glyceric acid	22.76	3TMS	00139
Fumaric acid	23.05	2TMS	00134
L-Serine	23.73	3TMS	00187
Pyrrole-2-carboxylic acid	24.32	2TMS	03094
L-Threonine	24.64	3TMS	00167
Aminomalonic acid	27.26	3TMS	01147
Malic acid	27.83	3TMS	00156
L-5-oxoproline	28.75	2TMS	00267
Threonic acid	29.72	4TMS	00943
Creatinine	29.84	3TMS	00562
L-Glutamine	31.68	3TMS	00641
D-Xylose	33.73	4TMS; 1MOX	00098
Ribitol	35.05	5TMS	00508
Glycerol 3-phosphate	35.92	4TMS	00126
Citric acid	37.37	4TMS	00094
Myristic acid (14:0)	37.55	1TMS	00806
L-Tyrosine	38.78	2TMS	00158
D-Fructose	38.99 39.25	5TMS; 1MOX 5TMS; 1MOX	00660
D-Glucose	39.42 39.66 40.11	5TMS 5TMS; 1MOX 5TMS; 1MOX	00122
D-Mannitol	40.65	6TMS	00765
L-Arabinofuranose	41.27	4TMS	12325
Palmitoleic acid ((9*Z*)-16:1)	41.90	1TMS	00207
D-Galactose	42.12	5TMS; 1MOX	00143
Palmitic acid (16:0)	42.36	1TMS	00220
*Scyllo*-Inositol	42.78	6TMS	06088
*Myo*-Inositol	44.21	6TMS	00211
Oleic acid ((9*Z*)-18:1)	46.24	1TMS	03229
Vaccenic acid ((11*E*)-18:1)	46.38	1TMS	03231
Stearic acid (18:0)	46.78	1TMS	00827
Mannitol 1-phosphate	49.13	7TMS	01530
D-Glucose-6-phosphate	49.35	6TMS	01401
(1-Myristoylglycerol)	49.92	2TMS	11561
Uridine	50.96	3TMS	00296
(1-Palmitoylglycerol)	52.56	2TMS	31074
(2-Stearoylglycerol)	54.33	2TMS	–
(1-Stearoylglycerol)	54.74	2TMS	31075
Cholesterol	60.71	1TMS	00067

**Notes:**

TMS, trimethylsilyl group; MOX, *O*-methoxyimine group.

HMDB, Human Metabolome Database (http://www.hmdb.ca/).

Metabolites in (parentheses) are contributed to by leaching from certain laboratory plasticware and were not included in the multivariate data analysis.

**Table 2:** Metabolites detected by GCMS in lysates of HMCL-7304 myotubes.

Metabolite	Retention time (min)	Derivatives formed	HMDB number
L-Lactic acid	13.33	2TMS	00190
Caproic acid	13.66	1TMS	00535
Glycolic acid	13.83	2TMS	00115
Hydroxylamine	15.22	3TMS	03338
L-Leucine	20.68	2TMS	00687
L-Isoleucine	21.42	2TMS	00172
L-Valine	18.74	2TMS	00883
Ethanolamine	20.48	3TMS	00149
Phosphate	20.81	3TMS	01429
L-Isoleucine	21.42	2TMS	00172
Glycine	21.84	3TMS	00123
Glyceric acid	22.76	3TMS	00139
L-Threonine	24.64	3TMS	00167
L-5-oxoproline	28.76	2TMS	00267
Glycerol 3-phosphate	35.92	4TMS	00126
Citric acid	37.37	4TMS	00094
Myristic acid (14:0)	37.57	1TMS	00806
D-Glucose	39.21 39.65 40.11	5TMS 5TMS; 1MOX 5TMS; 1MOX	00122
Palmitic acid (16:0)	42.36	1TMS	00220
*Myo*-Inositol	44.21	6TMS	00211
Oleic acid ((9*Z*)-18:1)	46.24	1TMS	03229
Vaccenic acid ((11*E*)-18:1)	46.39	1TMS	03231
Stearic acid (18:0)	46.78	1TMS	00827
(1-Myristoylglycerol)	49.93	2TMS	11561
(2-Palmitoylglycerol)	52.15	2TMS	–
(1-Palmitoylglycerol)	52.56	2TMS	31074
(2-Stearoylglycerol)	54.34	2TMS	–
(1-Stearoylglycerol)	54.75	2TMS	31075
Cholesterol	60.72	1TMS	00067

**Notes:**

TMS, trimethylsilyl group; MOX, *O*-methoxyimine group.

HMDB, Human Metabolome Database (http://www.hmdb.ca/).

Metabolites in (parentheses) are contributed to by leaching from certain laboratory plasticware and were not included in the multivariate data analysis.

**Figure 4:**
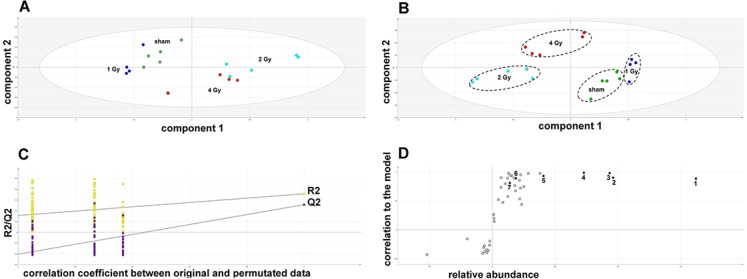
Metabolomic analysis on lysates of γ-irradiated HepG2 cells. (A) Unsupervised principal components analysis (PCA) scores plot showing separation of the sham irradiated (green symbols), 1 Gy γ-irradiated samples (blue symbols), the 2 Gy γ-irradiated (turquoise symbols) and 4 Gy γ-irradiated samples (red symbols). There was one outlier from the Hotelling’s T2 ellipse (95% confidence interval), belonging to the 1 Gy group, which has been removed. (B) Supervised projection to latent structures-discriminant analysis (PLS-DA) scores plot showing a clear separation of the sham, 1 Gy, 2 Gy and 4 Gy γ-irradiated samples. (C) Validation of the PLS-DA model using 100 iterations. The data are not over-modelled because the correlation coefficient (R2) fell below 0.3 and the predictability coefficient (Q2) fell below zero after data permutation. (D) Orthogonal PLS-DA (OPLS-DA) loadings S-plot for sham *versus* 2 Gy γ-irradiation of HepG2 cells. Each symbol represents a metabolite and its contribution to the separation between sham and 2 Gy γ-irradiated samples in panel B. Filled symbols are those metabolites selected for univariate statistical analysis. 1 = D-glucose; 2 = L-lactate; 3 = L-5-oxoproline; 4 = L-alanine; 5 = D-fructose; 6 = D-glucose 6-phosphate; 7 = succinate.

**Figure 5:**
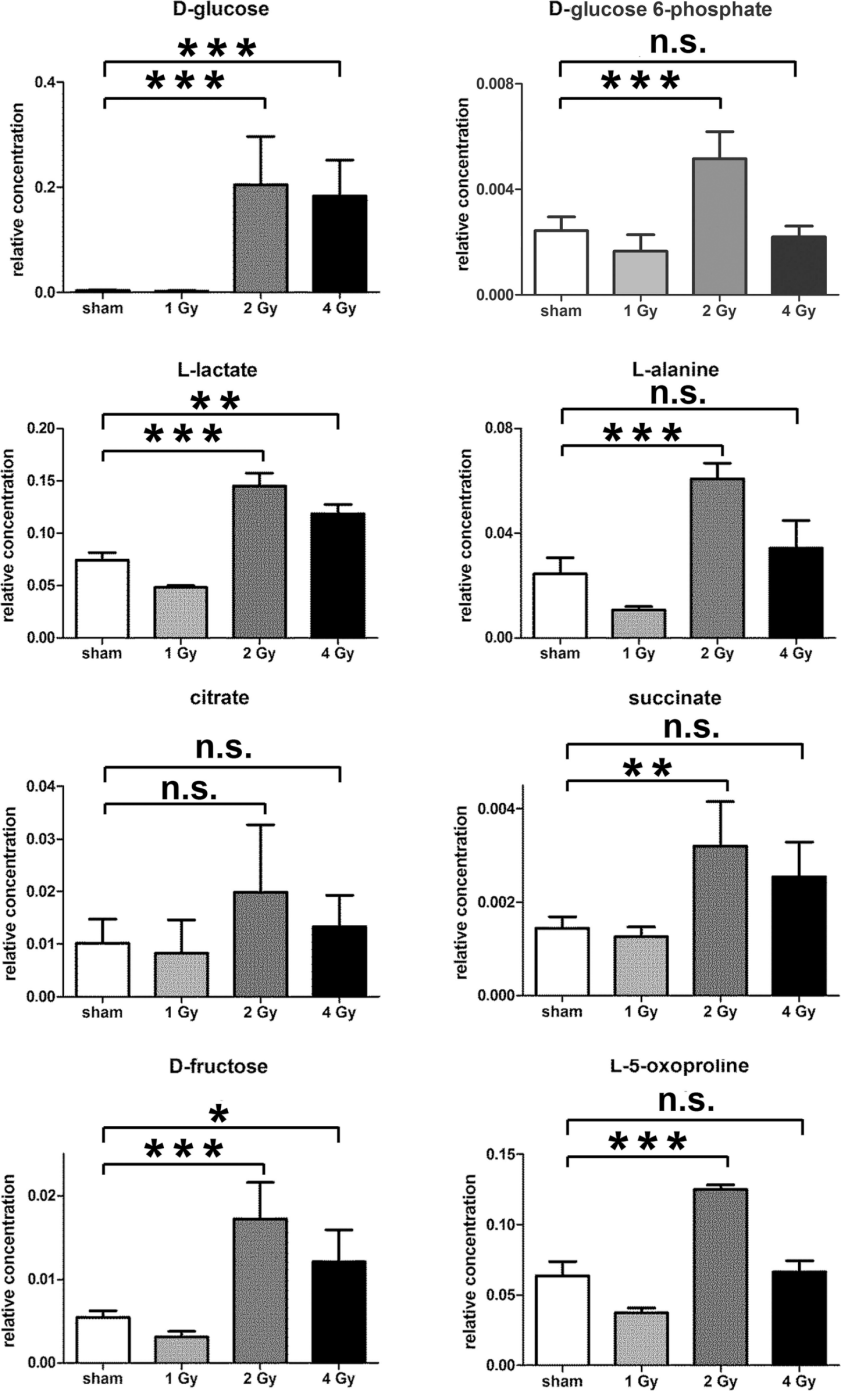
Univariate data analysis on metabolomic markers of γ-irradiated HepG2 cells. Data are presented as means ± standard deviation (S.D.). Data were analyzed by one-way ANOVA for each metabolite with Bonferroni’s correction for multiple comparisons. ANOVA was significant at P < 0.0001. There were no statistically significant differences between the sham irradiated and 1 Gy γ-irradiated samples for any metabolite. * means P < 0.05; ** means P < 0.01; *** means P < 0.001; n.s. means not statistically significant. Relative concentrations were calculated as the peak area of each metabolite divided by the peak area of the internal standard and are based upon 4 × 10^6^ cells.

The GCMS data from 20 cultures of HepG2 cells and 12 cultures of HMCL-7304 myotubes were subjected to multivariate data analysis, first by PCA, which shows the internal structure of the dataset and identifies any outliers. Figure 4A shows the PCA scores plot for γ-irradiated HepG2 cell lysates, with clustering of the sham, 1 Gy, 2 Gy and 4 Gy flasks. When a PLS-DA model was built for these data (Fig. 4B), the sham, 1 Gy, 2 Gy and 4 Gy clusters were all resolved. In order to check that the supervised analysis had not over-fitted the data, a leave-one-out cross-validation was performed with 100 permutations (Fig. 4C). Because the permuted correlation (R2) and predictability (Q2) fell below 0.3 and 0, respectively, the data were not over-fitted and the PLS-DA model was therefore valid. An OPLS-DA loadings S-plot was then employed to detect the upregulated metabolites in HepG2 cell lysates after 2 Gy γ-irradiation (Fig. 4D). D-Glucose (1), L-lactate (2), L-5-oxoproline (3), L-alanine (4), D-fructose (5), D-glucose 6-phosphate (6) and succinate (7) were all considered as candidates for upregulated metabolites and, together with citrate, were subjected to univariate statistics. Figure 5 shows that 2 Gy γ-irradiation of HepG2 cells led to a highly statistically significant (P < 0.001) increase in intracellular L-lactate, L-alanine, D-glucose, D-glucose 6-phosphate, D-fructose and L-5-oxoproline. This metabolic pattern is consistent with increased glucose mobilization/uptake in concert with elevated aerobic glycolysis (the Warburg effect), leading to increased synthesis of glucose 6-phosphate, lactate and alanine, the last two both from pyruvate. Increased fructose might represent shunting of excess glucose through the polyol pathway, which leads to fructose synthesis via sorbitol (Bjornstad et al., 2015), but fructose could equally be produced by other mechanisms, such as glucose 6-phosphate isomerase in the glycolytic pathway. In addition, the elevated 5-oxoproline is a sign of increased GSH turnover in response to oxidative stress (Emmett, 2014), but could potentially arise from other sources. In general, these effects were less pronounced after 4 Gy γ-irradiation of HepG2 cells. Changes in the TCA cycle intermediates citrate and succinate were of lower statistical significance or insignificant after HepG2 cell irradiation.

**Figure 6:**
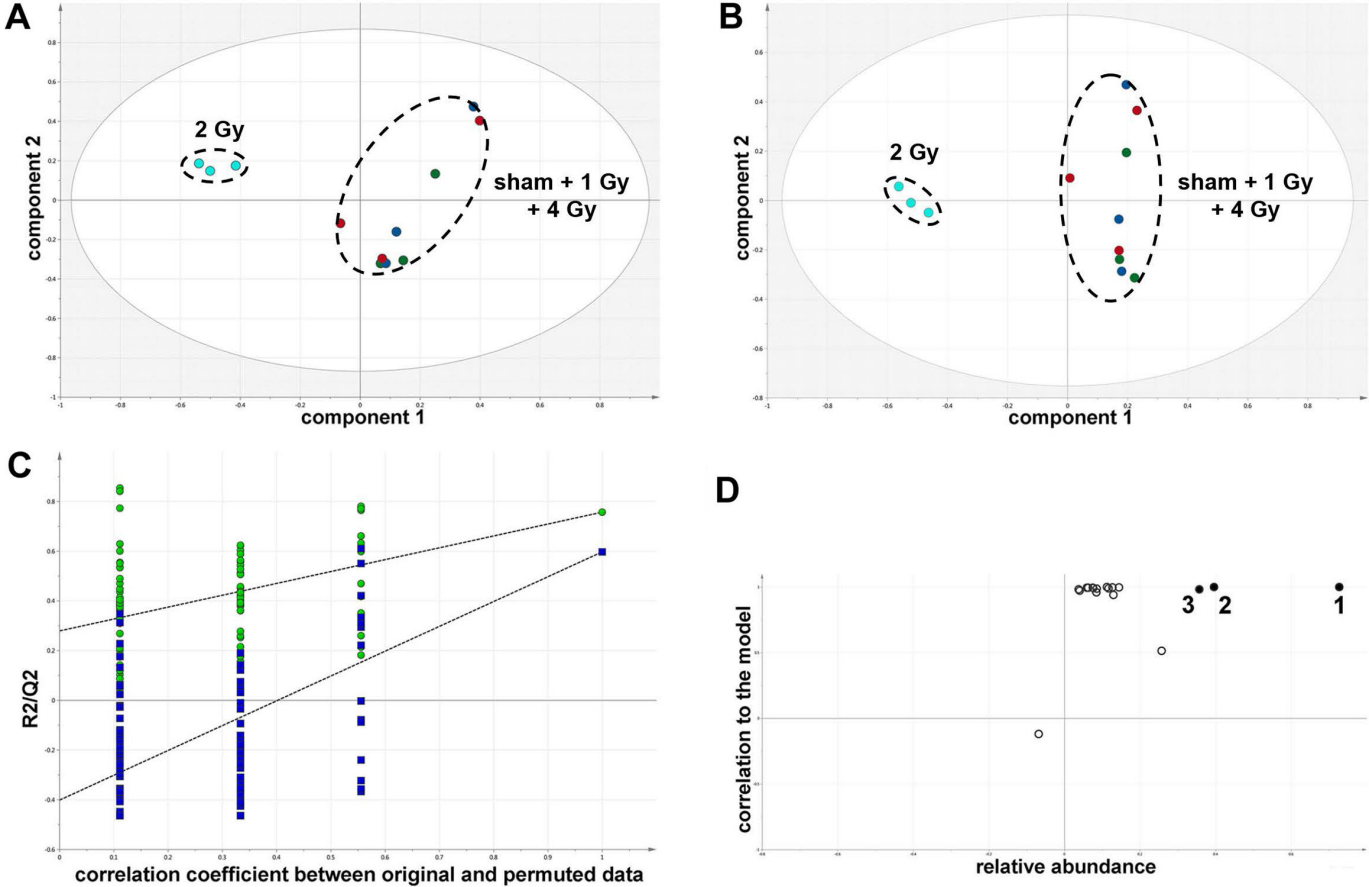
Metabolomic analysis on lysates of γ-irradiated HMCL-7304 cells. (A) Unsupervised PCA scores plot showing a clustering of the sham irradiated (green symbols), 1 Gy γ-irradiated samples (blue symbols) and 4 Gy γ-irradiated samples (red symbols) with the clear separation from the 2 Gy γ-irradiated (turquoise symbols). There were no outliers from the Hotelling’s T2 ellipse (95% confidence interval). (B) Supervised PLS-DA scores plot showing a clustering of the sham irradiated (green symbols), 1 Gy γ-irradiated samples (blue symbols) and 4 Gy γ-irradiated samples (red symbols) with the clear separation from the 2 Gy γ-irradiated (turquoise symbols). As for the PCA analysis, there were no outliers. (C) Validation of the PLS-DA model using 100 iterations. The data are not over-modelled because the correlation coefficient (R2) fell below 0.3 and the predictability coefficient (Q2) fell below zero after data permutation. (D) OPLS-DA loadings S-plot for sham *versus* 2 Gy γ-irradiation of HMCL-7304 cells. Each symbol represents a metabolite and its contribution to the separation between sham and 2 Gy γ-irradiated samples in panel (B). Filled symbols are those metabolites selected for univariate statistical analysis. 1 = phosphate; 2 = D-glucose; 3 = L-lactate. All metabolites measured in HMCL-7304 cells were correlated significantly to the OPLS-DA model, except ethanolamine and L-5-oxoproline.

**Figure 7:**
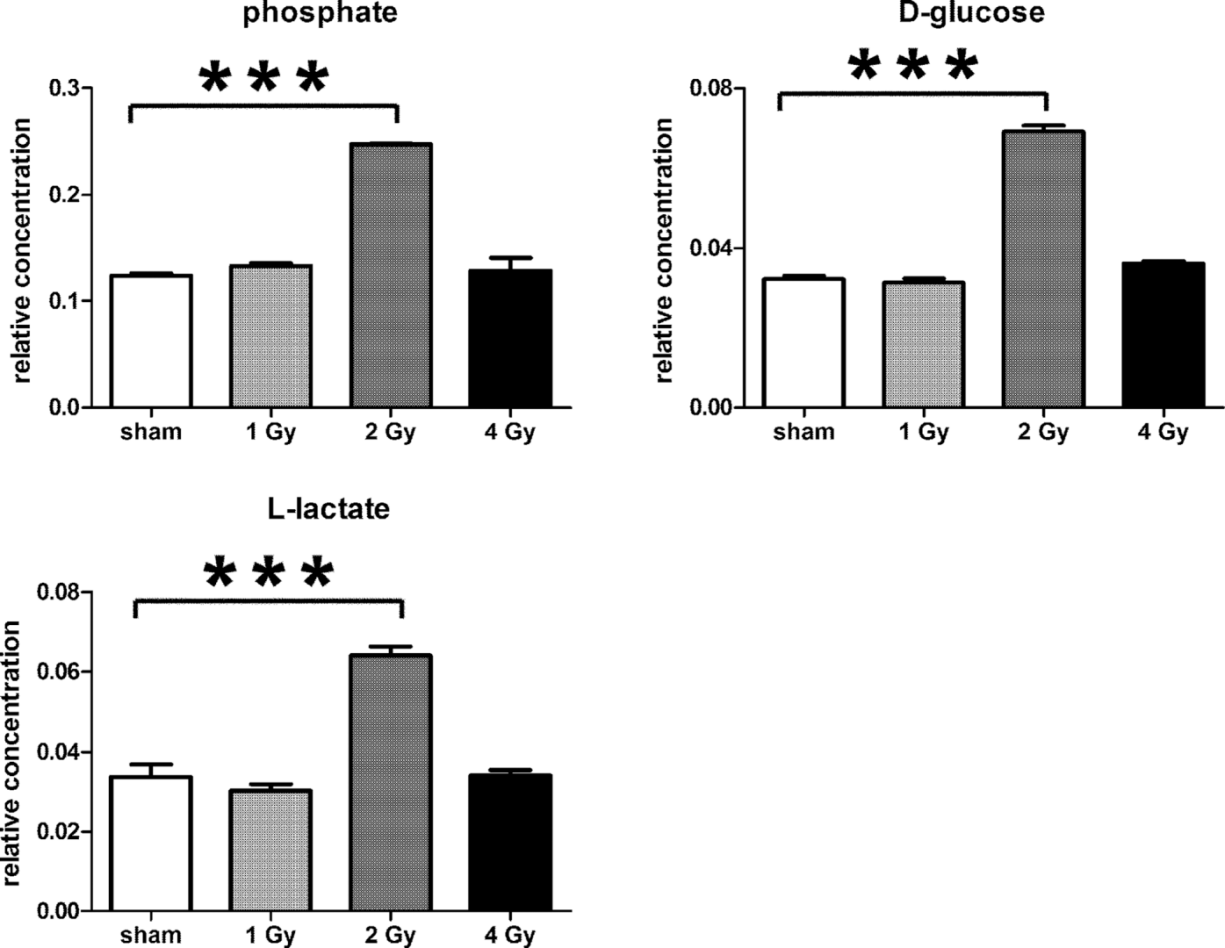
Univariate data analysis on metabolomic markers of γ-irradiated HMCL-7304. Data are presented as means ± S.D. Data were analyzed by one-way ANOVA for each metabolite with Bonferroni’s correction for multiple comparisons. ANOVA was significant at P < 0.0001. There were no statistically significant differences between the sham irradiated and either the 1 Gy γ-irradiated or 4 Gy γ-irradiated samples for any metabolite. *** means P < 0.001. Relative concentrations were calculated as the peak area of each metabolite divided by the peak area of the internal standard and are based upon 4 × 10^6^ cells.

In summary, the pattern of metabolic reprogramming observed in both cell lines resembled that of enhanced aerobic glycolysis, with elevated intracellular glucose and lactate in both hepatoma and muscle cells, together with elevated intracellular glucose 6-phosphate and alanine in HepG2 cells. In addition, there was evidence from increased intracellular 5-oxoproline of increased GSH turnover in HepG2. Such a finding is consistent with enhanced ROS production due to γ-irradiation. These effects were not observed at all radiation doses employed. In HepG2 cells, 1 Gy γ-irradiation had no observable effect (Fig. 5). The maximum response was observed at a dose of 2 Gy (glucose↑ ∼50-fold, P < 0.001; glucose 6-phosphate↑ 2-fold, P < 0.001; lactate↑ 2-fold, P < 0.001; alanine↑ 2.5-fold, P < 0.001), with a lower but statistically significant response at 4 Gy (glucose↑ ∼40-fold, P < 0.001; lactate↑ 1.6-fold, P < 0.01). In HMCL-7304 myotubes, the metabolic effect of γ-irradiation was much less pronounced and with an intracellular rise in glucose (2-fold, P < 0.001) and lactate (2-fold, P < 0.001) only observed at 2 Gy, with doses of 1 and 4 Gy indistinguishable from sham irradiation (Figs. 6 and 7).

## Discussion

Understanding the metabolic response of tissues to ionizing radiation is essential to the evaluation of the origin of metabolomic biomarkers for radiation exposure and to the development of novel treatment strategies for radiation sickness. To date, work has largely been conducted in laboratory animals, including mice, rats and rhesus monkeys, with almost no comprehension of the metabolomic response to radiation of cultured cells. One study has been reported in which human TK6 lymphoblastoid cells and the BJ fibroblast cell line were γ-irradiated and intracellular metabolites analyzed by ultra-performance liquid chromatography-electrospray ionization-quadrupole time-of-flight mass spectrometry (UPLC-ESI-QTOFMS) (Patterson et al., 2008). The OPLS-DA loadings S-plot revealed several metabolites upregulated by γ-irradiation, but none was identified. However, some intracellular metabolites were quenched by γ-irradiation and these were identified mainly as GSH, AMP and NAD^+^. Interestingly and contrary to the finding reported here, cellular 5-oxoproline was diminished in irradiated cells (Patterson et al., 2008). In this study, we sought to identify intracellular metabolites that were enhanced after γ-irradiation and chose GCMS-based metabolomics as a means to identify them. GCMS is better suited than simple reverse-phase UPLC-ESI-QTOFMS to the detection of small polar intermediate metabolites because these metabolites, such as amino acids and sugars, elute too early on UPLC (Patterson et al., 2011), making them difficult to quantitate.

The principal finding in irradiated HepG2 cells relative to sham-irradiated cells was a huge increase in intracellular glucose, with a concomitant elevation of glucose 6-phosphate and the two pyruvate metabolites lactate and alanine, suggesting enhancement of cytosolic aerobic glycolysis. As has been observed in hyperglycaemia, increased glycolytic flux leads to a saturation of hexokinase, followed by shunting of as much as one-third of glucose through aldose reductase in the polyol pathway, which uses one mole of NADPH per mole glucose metabolized and generates one mole of NADH per mole of fructose produced from the intermediate sorbitol (Yabe-Nishimura, 1998). GSH is a principal defense against oxidative stress in the liver and recycling of oxidized glutathione to GSH is carried out by glutathione reductase, for which NADPH is an obligatory cofactor. The reciprocal relationship between GSH and ROS has been demonstrated in HepG2 cells (Kim et al., 2010). In addition, when a transgenic mouse with a disrupted polyol pathway (Ho et al., 2000) was rendered short-term diabetic with streptozotocin-induced superoxide formation, GSH depletion and subsequent DNA damage, as seen in similarly treated wild-type mice, was not observed (Ho et al., 2006). Therefore, there is a clear relationship between shunting of excess glucose through the polyol pathway, GSH depletion and DNA damage. However, we have no evidence that the polyol pathway was activated in our irradiated cells, simply the conjecture that the excess glucose load makes it possible that some additional glucose is handled by aldose reductase and the polyol pathway.

In γ-irradiated HMCL-7304 myotubes, a simpler but similar metabolic pattern was observed, with elevated intracellular glucose and lactate. Unlike HepG2, this cell line was not derived from cancer cells, but rather from skeletal muscle of a 19 year-old healthy donor and a cell line developed by retroviral knock-in of the *TERT* and *CDK4* genes (Rokach et al., 2013). These cells are therefore not expected to display significant aerobic glycolysis. However, after γ-irradiation, HMCL-7304 myotubes plainly demonstrated the metabolic fingerprint of upregulated aerobic glycolysis, with increased intracellular glucose and enhanced lactate production.

The potential augmentation of the Warburg effect was not observed at all radiation doses. In both cell lines, there was no statistically significant difference between the sham-irradiated and the 1 Gy dose, indicating a clear threshold for putative upregulation of aerobic glycolysis by γ-irradiation. For HepG2 cells, both 2 Gy and 4 Gy stimulated large and highly statistically significant increases in intracellular glucose, but the increases in both lactate and alanine were more pronounced at 2 Gy compared to 4 Gy. In contrast, only 2 Gy γ-irradiation stimulated assumed aerobic glycolysis in HMCL-7304 myotubes. Therefore, it would appear that the highest radiation dose reduced the magnitude of the metabolic reprogramming. This may be due to increased genotoxicity at the 4 Gy dose, since γ-irradiation of FL5.12 cells with a dose of 20 Gy, resulted in decreases in both glucose uptake and glycolysis 24 h after irradiation (Zhou, Vander Heiden & Rudin, 2002). We also observed a high proportion of cells (∼47%) in G2/M at the highest radiation dose. It has recently been reported that human cervical carcinoma SiHa cells, both wild-type and a sub-clone with impaired mitochondrial respiration, moved from 21–31% to 46–47% in G2/M 24 h after a dose of 5 Gy γ-irradiation (Bol et al., 2015). Our findings, both of the dose-response relationship with respect to a possible Warburg effect, together with cell cycle behaviour, are consistent with the reports of others.

Our contention that γ-irradiation augmented cytosolic aerobic glycolysis in two cell lines derived from liver and muscle may have implications for the prevention or amelioration of tissue toxicity after exposure to ionizing radiation. This would be especially pertinent in cases where vast numbers of persons were exposed to varying doses, for example, in the case of a terrorist incident involving either a radiation or a nuclear device. Ionizing radiation generates hydroxyl radicals through radiolysis of intracellular water and these highly-reactive and short-lived species lead to a state of oxidative stress that has been estimated to be responsible for 70% of the tissue damage that results from ionizing radiation (Anuranjani & Bala, 2014). The metabolic reprogramming we observed in HepG2 cells caused a huge rise in intracellular glucose. It is likely that this may result in a flux of glucose through aldose reductase and the polyol pathway, with a resulting decrement of NADPH and thus depleted glutathione reductase activity. This will also compromise GSH production and the defense against oxidative stress, leading to enhanced tissue damage. If confirmed, these findings provide clues to new potential therapeutic and preventative interventions for radiation sickness.

## Conclusions

It is concluded from metabolomic profiles that γ-irradiation of human cell lines derived from liver and striated muscle causes a metabolic reprogramming consistent with amplified cytosolic aerobic glycolysis. Further investigation of this putative radiation-induced Warburg effect may lead to novel prevention and treatment strategies for radiation-induced tissue damage.

## Additional Information and Declarations

### Data Deposition

The following information was supplied regarding data availability:
http://dx.doi.org/10.6084/m9.figshare.1529823

http://dx.doi.org/10.6084/m9.figshare.1529824
